# Risk factors for intracranial infection after craniotomy: A case–control study

**DOI:** 10.1002/brb3.1658

**Published:** 2020-05-18

**Authors:** Li‐Yi Wang, Xu‐Hua Cao, Li‐Ke Shi, Zhi‐Zhao Ma, Yue Wang, Yan Liu

**Affiliations:** ^1^ Hospital Infection‐Control Department The Second Hospital of Hebei Medical University Shijiazhuang China; ^2^ Hospital Neurosurgery Department The Second Hospital of Hebei Medical University Shijiazhuang China

**Keywords:** craniotomy, intracranial infection, postoperative oral infection, risk factor, surgical season

## Abstract

**Background:**

Intracranial infection, serving as a severe postoperative infection after craniotomy, poses significant problems for patients' outcomes.

**Objective:**

To explore risk factors for intracranial infection after craniotomy.

**Methods:**

A total of 2,174 patients who underwent craniotomy from 1 May 2018 to 30 June 2019 were retrospectively studied. Finally, 196 patients with intracranial infections were classified as case group, and 392 patients randomly selected from patients without intracranial infection were classified as control group. Demographic, clinical, laboratory, microbiological, and antimicrobial data were systemically recorded. The characteristics, pre‐ and postoperative variables, and other variables were evaluated as risk factors for intracranial infection by univariate analysis and binary logistic regression model.

**Results:**

There was no significant difference in terms of demographics between two groups, except for gender, hypertension, length of stay (LOS), intraoperative blood loss, tumor, and trauma surgery. The independent risk factors were male, age ≤45, hypertension, tumor surgery, surgery in autumn (compared with spring), surgical duration ≥4 hr, intraoperative blood loss ≥400 ml, and postoperative oral infection, coma, and serum RBC > normal value. Trauma surgery (*p* < .001, OR = 0.05, 95% CI: 0.017–0.144) was an independent protective factor (*p* < .05, OR < 1) for intracranial infection. All 196 patients in the case group submitted specimens for cerebrospinal fluid (CSF) cultures, and 70 (35.71%) patients had positive results. Gram‐positive pathogens predominated (59 cases, 84.28%). *Staphylococcus* were the most common causative pathogens, and fully resistant to aztreonam, cefazolin, and benzylpenicillin, but not resistant to linezolid and minocycline.

**Conclusion:**

Identifying the risk factors, pathogens, and pathogens' antibiotic resistance for intracranial infection after craniotomy plays an important role in the prognosis of patients.

## Graphical abstract

In our study, surgical season and postoperative oral infection are firstly determined as the new risk factors for intracranial infection. At the same time, microorganisms and its antibiotic resistance isolated from intracranial infections were determined in the present study.

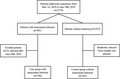

## 1 INTRODUCTION

Craniotomy, as a neurosurgical procedure, has been performed more than a century and is characterized by performing within the intracranial space (Adaaquah, Gates, & Van Gompel, 2018; Gonzalez‐Darder, 2016). Intracranial infections, including brain abscess, meningitis, and subdural or epidural infections, are serious complications after craniotomy (Horan, Andrus, & Dudeck, 2008). Intracranial infection poses significant problems for patients' outcomes (Kourbeti, Jacobs, Koslow, Karabetsos, & Holzman, 2007; National Nosocomial Infections Surveillance System, 2004), and it may cause high rate of morbidity and mortality, prolonged length of stay, extra healthcare cost, and multiple surgeries (Hweidi, Barbarawi, Tawalbeh, Al‐Hassan, & Al‐Ibraheem, 2018; Korinek, 1997; Rebuck, Murry, Rhoney, Michael, & Coplin, 2000).

White blood cell count or neutrophil count is still indicators of infection and clinical judgment; however, their specificity might not be high (Folyovich et al., 2014; Westendorp, Nederkoorn, Vermeij, Dijkgraaf, & van de Beek, 2011). Surgical drainage and antibiotics are also the effective therapy to treat intracranial infection (Dashti et al., 2008; Kural et al., 2019). However, many drugs are facing difficulty to penetrate into brain due to the blood–brain barrier (BBB) (Daneman & Prat, 2015). Meanwhile, cerebrospinal fluid (CSF) only can be drained a little through lumbar puncture. Therefore, it is of great significance to explore risk factors for intracranial infection after craniotomy in order to timely prevent it.

Currently, more than 10 high‐risk factors have been reported, which were mainly for surgical site infection after craniotomy (Adaaquah et al., 2018; Fang, Zhu, Zhang, Xia, & Sun, 2017; Kourbeti et al., 2007). However, the risk factors of season and postoperative oral infection were not analyzed. In the 1982s, the seasonal variation in arterial blood pressure was reported (Brennan, Greenberg, Miall, & Thompson, 1982). In addition, Herweh et al have concluded that hypertensive intracerebral hemorrhage was associated with the increased air pressure by conducting a worldwide cohort (Herweh et al., 2017). As regards the oral infection, one previous study has reported that brain abscesses might be the potential deadly complications of odontogenic infections through summarizing related cases (Moazzam, Rajagopal, Sedghizadeh, Zada, & Habibian, 2015). However, there was no case–control study performed to verify the relationship between oral infection and intracranial infection.

In our study, we calculated the prevalence of intracranial infection after craniotomy and further identified the risk factors for intracranial infection, especially some new risk factors such as surgical season and postoperative oral infection. Furthermore, microorganisms and its antibiotic resistance isolated from cerebrospinal fluid (CSF) were also determined.

## 2 PARTICIPANTS AND METHODS

### 2.1 Participants

The enrolled patients were admitted from Department of Neurosurgery in the Second Hospital of Hebei Medical University between 1 May 2018 and 30 June 2019. Written informed consent was obtained from all individual participants. This study was approved by the Research Ethics Committee of the Second Hospital of Hebei Medical University (No. 2018‐R084). Patients who underwent craniotomy were included. Patients were excluded if they: (a) did not receive craniotomy; (b) were accompanied by severe organ functional lesion, malignant tumor, metabolic diseases, blood systemic diseases, and spinal deformity; (c) gave up treatment; (d) were too fat to perform lumbar puncture; (e) had failed lumbar puncture; (f) were treated by nonsurgical treatment, such as intravenous antibiotics; and (g) had adhesion of subarachnoid space to cerebrospinal fluid circulation disorder.

### 2.2 Study design

**FIGURE 1:**
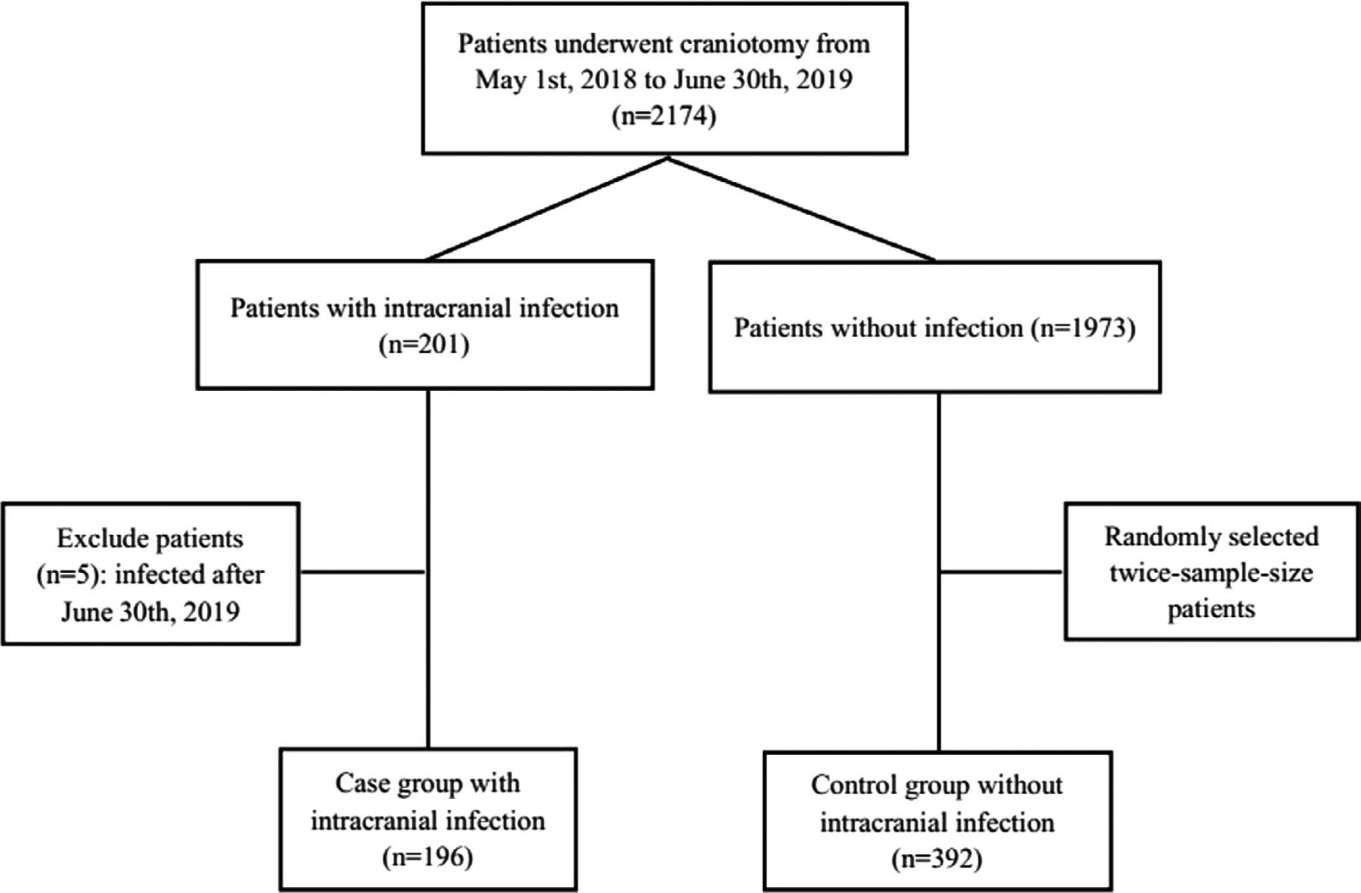
Flow diagram of patient selection

This retrospective case–control study was performed according to clinical test procedures of National Health and Family Planning Commission of the People's Republic of China. A total of 2,174 consecutive patients who underwent craniotomy were enrolled. Intracranial infection was determined by two brain surgery experts based on the indicators of the cases and the results of biochemical microorganisms. If the two experts got different decisions, the third expert would control quality by interpreting the results. At the same time, patients in the case groups were identified to have consecutive intracranial infections during hospitalization. Among the 2,174 patients, 201 patients were infected with intracranial infection after craniotomy, but five patients were excluded for having intracranial infection after 30 June 2019. Therefore, the 196 patients with intracranial infections were classified as case group. The data collection was difficult due to the poor condition of our hospital, and 392 cases in the control group, twice as many as in the case group, were randomly selected from the remaining 1,973 patients without intracranial infection. Case and control groups chose the same consecutive patients who underwent craniotomy. The flow diagram is shown in Figure 1. The prevalence of nosocomial intracranial infection was defined to be happened after 48 hr of admission to control the prevalence–incidence bias. In addition, the blind method was used to control the investigation bias, all patients or clinicians did not know the information to distinguish the case group from the control group.

### 2.3 Definitions

Intracranial infection was diagnosed according to the definitions of Centers for Disease Control (CDC) (Horan et al., 2008). The prevalence of intracranial infection was defined to be happened: (a) after 48 hr of admission; (b) during operation or pathological examination; (c) from organisms cultured from brain tissue or dura; (d) considering at least the following two signs such as dizziness, fever (>38°C), headache, local neurosis, consciousness‐changing, or confusion; (e) and from (a) microorganisms identified from brain tissue, abscess tissue, blood, or urine; (b) diagnostic single‐antibody titer (IgM) or fourfold increase in paired sera (IgG) for pathogen; and (c) radiographic evidence of infection.

The surgical seasons were divided into four according to the 24 solar terms of China. Spring was from 15 April to 20 June in 2018 and 21 March to 15 April in 2019. Summer was defined from 21 June to 22 September in 2018. Autumn was from 23 September to 6 November in 2018. Winter was defined from 7 November in 2018 to 20 March in 2019.

Cerebrospinal fluid leak was defined as any leak of the fluid that surrounds the brain and spinal cord and escapes from the cavities within the brain or central canal in the spinal cord (Abuabara, 2007), and was reported to be associated with the development of meningitis (Jones & Becker, 2001).

### 2.4 Data collection

The demographic, clinical, laboratory, microbiological, and antimicrobial data were systemically analyzed by same team, laboratory and healthcare department. The clinical data were further analyzed by reviewing electronic medical records and files. The other data were collected from three electronic surveillance systems: Nosocomial infection records were from the Ongoing Nosocomial Infection Surveillance of Xinglin; clinical data were from electronic medical records; and microbiological and antimicrobial data were from microbial systems.

The detailed demographic, clinical, and laboratory factors were as follows: Preoperative factors included hospital length of stay (LOS), emergency (patients with serious condition or suffering from car accident), and other surgeries (surgery with posterior fossa, bilateral surgery, and external CSF drainage); intraoperative factors included surgical season, surgical duration, and intraoperative blood loss; and postoperative factors included signs of oral infection, cerebral hernia, acid inhibitors, reoperation, CSF leak, coma, intensive nursing care, American Society of Anesthesiologists (ASA) score, albumin level (ALB), high‐sensitivity C‐reactive protein (hsCRP) level, red blood cell count (RBC) level, and serum hemoglobin (HGB) level. The normal values of postoperative indexes of ALB, hsCRP, RBC, and serum HGB were 40–55 g/L, 0–6 mg/L, 4.3–5.8 × 10^12^ L (men) and 3.8–5.10 × 10^12^ L (women), and 130–175 g/L (men) and 115–150 g/L (women), respectively. In addition, gender, age, hypertension, hyperlipidemia, diabetes mellitus, trauma, and tumor were also included.

### 2.5 Determination of the cut‐point of quantitative variables

As shown in Table 1, the average age of the targeted population was 48.87 ± 16.21 years. The age of 45 years was selected as cut‐point. The average surgical duration and preoperative LOS were 4.62 ± 2.03 hr and 7.44 ± 6.56 days, 4 hr and 7 days were selected as cut‐points, respectively. The ASA score ranges from 1 to 5 (Saklad, 1941). The mean ASA score in the case and control group was 2.49 ± 0.89 and 2.51 ± 0.85, respectively, a score of 2 was selected as cut‐points. Due to intraoperative blood loss, at least 400 ml was considered as a major bleeding in clinical diagnosis, 400 ml was selected as cut‐points.

**TABLE 1:** Baseline characteristics of the case group and the control group

Variable	Targeted population (*n* = 588)	Case group (*n* = 196)	Control group (*n* = 392)	*p* (case vs. control)
Age (years, mean [*SD*])	48.87 ± 16.21	47.08 ± 17.10	49.76 ± 15.69	.092
Gender (men, *n* [%])	290 (49.32)	109 (55.61%)	181 (46.17%)	.031
Surgical duration (hr, mean [*SD*])	4.62 ± 2.03	5.46 ± 2.27	4.19 ± 1.75	.439
Preoperative LOS (days, mean [*SD*])	7.44 ± 6.56	7.76 ± 6.90	7.28 ± 6.39	.404
LOS (days, mean [*SD*])	27.08 ± 16.95	35.45 ± 21.14	22.89 ± 12.49	<.001
Intraoperative blood loss (ml, median [IQR])	200.00 (100.00 – 400.00)	300.00 (150.00, 475.00)	200.00 (100.00, 300.00)	<.001
Postoperative ASA score	2.50 ± 0.86	2.49 ± 0.89	2.51 ± 0.85	.731
Hypertension (%)	243 (41.33)	93 (47.45)	150 (38.26)	.033
Hyperlipidemia (%)	3 (0.51)	0 (0.00)	3 (0.77)	.219
Diabetes mellitus (%)	59 (10.03)	21 (10.71)	38 (9.69)	.698
Trauma surgery (%)	81 (13.78)	7 (3.57)	74 (18.88)	<.001
Tumor surgery (%)	185 (31.46)	91 (46.43)	94 (23.98)	<.001
Postoperative serum RBC (1,012 L, mean [*SD*])	4.27 ± 4.23	3.99 (3.60, 4.31)	3.97 ± 0.58	.162
Postoperative serum HGB (g/L, mean [*SD*])	118.98 ± 19.98	120.03 ± 23.84	118.45 ± 17.75	.136
Postoperative serum hsCRP (mg/L, median [IQR])	13.250 (6.500 – 32.52)	13.40 (6.10, 30.36)	11.80 (6.68, 37.10)	.376
Postoperative serum ALB (g/L, mean [*SD*])	38.166 ± 16.207	39.42 ± 16.00	37.54 ± 16.30	.186

Abbreviations: ALB, albumin; ASA, American Society of Anesthesiologists; HGB, hemoglobin; hsCRP, high‐sensitivity C‐reactive protein; IQR, interquartile range; LOS, length of stay; RBC, red blood cell count; *SD*, standard deviation.

### 2.6 The clinical diagnostic routine of microbiology methods

If patients in the case group were with the symptoms of headaches, fever, nausea, and vomiting, 3–5 ml CSF was collected from brain or abscess tissue by needle aspiration or biopsy during surgical operation or autopsy, and stored in sterile test tubes for detecting the infection within 1 hr. The microorganisms were cultured in the Vitek compact autokinetic microbe culture instrument (bioMerieux).

### 2.7 The clinical diagnostic routine of antimicrobial susceptibility testing

Antimicrobial susceptibility test was implemented through computer‐assisted microbiology laboratory database. Thirty‐four antimicrobial agents were tested including aztreonam, cefazolin, benzylpenicillin, cefuroxime, penicillin, ampicillin, ampicillin/sulbactam, ceftriaxone, erythromycin, biapenem, cefotetan, cefepime, piperacillin, cefoxitin, clindamycin, meropenem, imipenem, ceftazidime, tobramycin, piperacillin/tazobactam, ciprofloxacin, levofloxacin, cefoperazone/sulbactam, polymyxin B, tetracycline, gentamicin, moxifloxacin, amikacin, nitrofurantoin, rifampicin, vancomycin, tigecycline, linezolid and minocycline. Polymyxin was performed by the broth dilution method to determine the minimum inhibitory concentrations (MICs), while others were determined by the agar dilution method (Wiegand, Hilpert, & Hancock, 2008). The quality control strains were *Escherichia coli* ATCC 25922, *Pseudomonas aeruginosa* ATCC 27853, *Staphylococcus aureus* ATCC 29213, and *Enterococcus faecalis* ATCC 29212. The sensitive or intermediary strains were defined as nonresistant strains in the antimicrobial susceptibility testing. All results of antimicrobial susceptibility test were interpreted according to the updated standards recommended by Clinical and Laboratory Standard Institution (CLSI) and analyzed by Whonet 5.6 software (Hsueh et al., 2010; Nakamura et al., 2007).

### 2.8 Statistical analysis

Statistical analyses were performed using SPSS Statistics version 22.0 (IBM Corporation). Controls were randomly selected from patients without intracranial infection after craniotomy by SPSS Statistics version 22.0. The proportion of the case group versus control group was 1:2. Categorical variables were presented as frequencies and percentages. Normally and non‐normally distributed continuous variables were given as means ± standard deviation (*SD*) and median (interquartile range, IQR). Comparisons of continuous variables were performed using two‐sided *t* test for normally distributed variables or the chi‐square test for dichotomous variables. As for non‐normally distributed continuous variables, we used Wilcoxon rank sum test. The chi‐square test was used to screen potential risk factors, and independent risk factors for intracranial infection were determined based on binary logistic regression analysis. Intracranial infection was employed as dependent variable in logistic regression model to adjusting the confounder. Variables with *p* value < .10 tested by univariate analysis were enrolled in binary logistic regression analysis. For all statistical data in binary logistic regression analysis, variables with *p* < .05 were significant. EpiData 3.1 was used to input these data by two graduate students. We have another staff to verify these data.

## 3 RESULT

### 3.1 Comparisons of demographics between the case group and the control group

The analysis included 588 patients, 196 cases in the case group and 392 cases in the control group. Men accounted for 49.32% of all patients. As shown in Table S1, the baseline between every kind of missing value group and nonmissing value group was comparable, with *p* value above .05. Then, the expectation–maximization (EM) in missing value analysis of SPSS 22.0 was used to replace missing values with an estimation. The *p* value of Little's MCAR test was .000, so the missing values were missing at random (MAR). The baseline characteristics of the case group and control group are summarized in Table 1. There was no significant difference in terms of age (47.08 ± 17.10 vs. 49.76 ± 15.69, *p* = .092), surgical duration (5.46 ± 2.27 vs. 4.19 ± 1.75, *p* = .439), preoperative LOS (7.76 ± 6.90 vs. 7.28 ± 6.39, *p* = .404), ASA score (2.49 ± 0.89 vs. 2.51 ± 0.85, *p* = .731), hyperlipidemia (0.00% vs. 0.77%, *p* = .219), diabetes mellitus (10.71% vs. 9.69%, *p* = .698), postoperative serum RBC [3.99 (3.60, 4.31) vs. (3.97 ± 0.58), *p* = .162], postoperative serum HGB (120.03 ± 23.84 vs. 118.45 ± 17.75, *p* = .136), postoperative serum hsCRP [13.40 (6.10, 30.36) vs. 11.80 (6.68, 37.10), *p* = .376], and postoperative serum ALB (39.42 ± 16.00 vs. 37.54 ± 16.30, *p* = .186) between the case group and the control group. However, there were significant differences in terms of gender (male, 55.61% vs. 46.17%, *p* = .031), LOS (35.45 ± 21.14 vs. 22.89 ± 12.49, *p* < .001), intraoperative blood loss [300.00 (150.00, 475.00) vs. 200.00 (100.00, 300.00), *p* < .001], hypertension (47.45% vs. 38.26%, *p* = .033), trauma surgery (3.57% vs. 18.88%, *p* < .001), and tumor surgery (46.43% vs. 23.98%, *p* < .001) between the case group and the control group.

### 3.2 Univariate analysis of potential risk factors

The potential risk factors are shown in Table 2. Men were more likely to have intracranial infection after craniotomy compared with women (*p* < .1). Age ≤ 45, hypertension, nontrauma surgery, and tumor surgery were significantly associated with an increased risk of intracranial infection (*p* < .1). Hyperlipidemia and diabetes mellitus were not the potential risk factors related to intracranial infection (*p* > .1).

**TABLE 2:** Univariate analysis of potential risk factors for intracranial infection after craniotomy

Variable (%)	Assigned	Case group (*n* = 196)	Control group (*n* = 392)	*χ* ^2^	*p*	OR	95% CI
Gender	Female	87 (44.39)	211 (53.83)	4.657	.031	1.461	1.035–2.062
	Male	109 (55.61)	181 (46.17)				
Age	>45	117 (59.69)	281 (71.68)	8.588	.003	1.709	1.192–2.450
	≤45	79 (40.31)	111 (28.32)				
Hypertension	No	103 (52.55)	242 (61.73)	4.545	.033	1.457	1.030–2.060
	Yes	93 (47.45)	150 (38.26)				
Hyperlipidemia	No	196 (100.00)	389 (99.23)	1.508	.219	0.665	0.628–0.704
	Yes	0 (0.00)	3 (0.77)				
Diabetes mellitus	No	175 (89.29)	354 (90.31)	0.151	.698	1.118	0.637–1.963
	Yes	21 (10.71)	38 (9.69)				
Trauma surgery	No	189 (96.43)	318 (81.12)	25.773	<.001	0.159	0.072–0.353
	Yes	7 (3.57)	74 (18.88)				
Tumor surgery	No	105 (53.57)	298 (76.02)	30.538	<.001	2.748	1.909–3.954
	Yes	91 (46.43)	94 (23.98)				
Preoperative LOS (days)	<7	94 (47.96)	193 (49.23)	0.085	.771	1.052	0.747–1.483
	≥7	102 (52.04)	199 (50.77)				
Emergency	No	138 (70.41)	274 (69.90)	0.016	.899	0.976	0.671–1.420
	Yes	58 (29.59)	118 (30.10)				
Surgery with posterior fossa	No	164 (83.67)	356 (90.82)	6.519	.011	1.930	1.158–3.216
	Yes	32 (16.33)	36 (9.18)				
Bilateral surgery	No	191 (97.45)	377 (96.17)	0.647	.421	0.658	0.236–1.837
	Yes	5 (2.55)	15 (3.83)				
External CSF drainage	No	1 (0.51)	0 (0.00)	2.003	.333	NA	NA
	Yes	195 (99.49)	392 (100.00)				
Surgical season	Spring	31 (15.82)	116 (29.59)	22.18	<.001	NA	NA
	Summer	58 (29.59)	128 (32.65)				
	Autumn	66 (33.67)	75 (19.13)				
	Winter	41 (20.92)	73 (18.62)				
Surgical duration (hr)	<4	54 (27.55)	196 (50.00)	26.98	<.001	2.630	1.815–3.810
	≥4	142 (72.45)	196 (50.00)				
Intraoperative blood loss (ml)	<400	123 (62.76)	306 (78.06)	15.51	<.001	2.112	1.451–3.074
	≥400	73 (37.24)	86 (21.94)				
Postoperative oral infection	No	191 (97.45)	390 (99.49)	4.627	.081	5.105	0.981–26.552
	Yes	5 (2.55)	2 (0.51)				
Postoperative cerebral hernia	No	190 (96.94)	388 (98.98)	3.255	.071	3.063	0.854–10.98
	Yes	6 (3.06)	4 (1.02)				
Postoperative acid inhibitors	No	62 (31.63)	163 (41.58)	5.475	.019	1.538	1.071–2.209
	Yes	134 (68.37)	229 (58.42)				
Postoperative ASA score	<2	15 (7.65)	19 (4.85)	1.889	.169	0.615	0.305–1.238
	≥2	181 (92.35)	373 (95.15)				
Reoperation	No	175 (89.29)	373 (95.66)	8.792	.003	2.647	1.362–5.143
	Yes	21 (10.71)	17 (4.34)				
Postoperative CSF leak	No	194 (98.98)	392 (100.00)	4.014	.111	NA	NA
	Yes	2 (1.02)	0 (0.00)				
Postoperative coma	No	159 (81.12)	345 (88.01)	5.063	.024	1.708	1.068–2.733
	Yes	37 (18.88)	47 (11.99)				
Postoperative intensive nursing care	No	183 (93.37)	361 (92.09)	0.307	.579	0.827	0.423–1.619
	Yes	124 (63.27)	203 (51.79)				
Postoperative ALB <40–55 g/L	No	52	85	1.718	.190	0.767	0.515–1.141
	Yes	144	307				
Postoperative ALB >40–55 g/L	No	189	389	4.591	.032	4.802	1.228–18.779
	Yes	7	3				
Postoperative hsCRP >0–6 mg/L	No	40	95	1.082	.298	1.247	0.822–1.893
	Yes	156	297				
Postoperative RBC < normal value	No	92	184	0.000	1.000	1.000	0.709–1.410
	Yes	104	208				
Postoperative RBC > normal value	No	189	388	4.632	.031	3.593	1.039–12.423
	Yes	7	4				
Postoperative HGB < normal value	No	95	190	0.000	1.000	1.000	0.710–1.409
	Yes	101	202				
Postoperative HGB > normal value	No	193	392	6.031	.037	NA	NA
	Yes	3	0				

Note

Normal value (postoperative RBC): the total number of normal men and women in the case group and control group, the normal value was 4.3–5.8 × 10^12^ L (men) and 3.8–5.10 × 10^12^ L (women); normal value (postoperative HGB): the total number of normal men and women in the case group and the control group, the normal value was 130–175 g/L (men) and 115–150 g/L (women).

Abbreviations: ALB, albumin; ASA, American Society of Anesthesiologists; CI, confidence interval; CSF, cerebrospinal fluid; HGB, hemoglobin; hsCRP, high‐sensitivity C‐reactive protein; LOS, length of stay; NA, no available value; OR, odds ratio; RBC, red blood cell count.

Surgery with posterior fossa, surgical season, surgical duration ≥4 hr, intraoperative blood loss ≥400 ml, postoperative oral infection, postoperative cerebral hernia, postoperative using acid inhibitors, reoperation, and postoperative coma were likely correlated with the development of intracranial infection (*p* < .1). However, preoperative hospital LOS ≥7 days, emergency, bilateral surgery, external CSF drainage, postoperative ASA score ≥2, postoperative CSF leak, and postoperative intensive nursing care were not the potential risk factors related to intracranial infection (*p* > .1).

Postoperative ALB > normal value, postoperative RBC > normal value, and postoperative serum HGB > normal value were potential risk factors for intracranial infection (*p* < .1), whereas postoperative ALB < normal value, postoperative hsCRP > normal value, postoperative RBC < normal value, and postoperative serum HGB < normal value were not the potential risk factors for intracranial infection (*p* > .1).

### 3.3 Binary regression analyses for intracranial infection

Male, age ≤45, hypertension, nontrauma surgery, tumor surgery, surgery with posterior fossa, surgical season, surgical duration ≥4 hr, intraoperative blood loss ≥400 ml, postoperative oral infection, postoperative cerebral hernia, postoperative acid inhibitors, reoperation, postoperative coma, and postoperative ALB > normal value, RBC > normal value, and serum HGB > normal value with *p* value lower to .1 were further entered into binary logistic regression model with conditional forward displayed at last step (Table S2). Intracranial infection was selected as dependent variable in binary logistic regression model. After adjustment, the factors with *p* ≥ .05, including surgery with posterior fossa, postoperative cerebral hernia, postoperative acid inhibitors, reoperation, postoperative ALB > normal value, and serum HGB > normal value, were excluded. Trauma surgery was retained as independent protective factor for intracranial infection, whereas the other 10 variables were retained as independent risk factors for intracranial infection (*p* < .05).

Men were 1.775 times (OR = 1.775, 95% CI: 1.185–2.660) likely to be infected with intracranial infection than women. Some factors were related to intracranial infection including age ≤45 (OR = 2.738, 95% CI: 1.737–4.318), hypertension (OR = 1.903, 95% CI: 1.225–2.957), tumor surgery (OR = 2.287, 95% CI: 1.476–3.545), surgical duration ≥4 hr (OR = 1.973, 95% CI: 1.251–3.113), intraoperative blood loss ≥400 ml (OR = 1.871, 95% CI: 1.167–3.001), postoperative oral infection (OR = 6.565, 95% CI: 1.084–39.771), postoperative coma (OR = 4.308, 95% CI: 2.136–8.689), and postoperative RBC > normal value (OR = 7.838, 95% CI: 1.833–33.507). Season was also an independent risk factor for intracranial infection. In addition, patients who underwent craniotomy in the autumn (*p* < .05, OR = 2.866, 95% CI: 1.592–5.159) were more susceptible to intracranial infection compared with those who underwent surgery in spring, which served as the baseline, while no relationship existed in other surgical seasons (*p* > .05). Patients who underwent surgery for trauma could led to corresponding 95.00% (OR = 0.050, 95% CI: 0.017–0.144) reduction of intracranial infection (Table 3).

**TABLE 3:** Binary logistic regression analysis for intracranial infections after craniotomy

Variable	Unadjusted OR	Unadjusted 95% CI	*B*	*S*_b_	Wald *χ* ^2^	*p*	OR	95% CI
Gender	1.461	1.035–2.062	0.574	0.206	7.752	.005	1.775	1.185–2.660
Age (years)	1.709	1.192–2.450	1.007	0.232	18.794	<.001	2.738	1.737–4.318
Hypertension	1.457	1.030–2.060	0.644	0.225	8.191	.004	1.903	1.225–2.957
Trauma surgery	0.159	0.072–0.353	−3.005	0.544	30.519	<.001	0.050	0.017–0.144
Tumor surgery	2.748	1.909–3.954	0.827	0.224	13.694	<.001	2.287	1.476–3.545
Season (spring)	NA	NA	NA	NA	14.004	.003	NA	NA
Season (summer)	1.696	1.025–2.505	0.271	0.288	0.883	.347	1.311	0.745–2.307
Season (autumn)	3.293	1.965–5.518	1.053	0.300	12.323	<.001	2.866	1.592–5.159
Season (winter)	2.102	1.212–3.645	0.513	0.316	2.632	.105	1.671	0.899–3.105
Surgical duration ≥4 hr	2.630	1.815–3.810	0.680	0.233	8.533	.003	1.973	1.251–3.113
Intraoperative blood loss ≥400 ml	2.112	1.451–3.074	0.627	0.241	6.768	.009	1.871	1.167–3.001
Postoperative oral infection	2.368	1.202–4.666	1.882	0.919	4.192	.041	6.565	1.084–39.771
Postoperative coma	2.647	1.362–5.143	1.460	0.358	16.650	<.001	4.308	2.136–8.689
Postoperative RBC > normal value	1.708	1.068–2.733	2.059	0.741	7.716	.005	7.838	1.833–33.507

Abbreviations: CI, confidence interval; OR, odds ratio; RBC, red blood cell count.

### 3.4 Distribution of microorganisms isolated from CSF of patients in the case group

All 196 patients in the case group with intracranial infection submitted specimens for CSF cultures, and 70 (35.71%) patients had positive results (Table 4). Gram‐positive pathogens predominated (59 cases, 84.28% of total positive cultures); among them, Coccus were the most common pathogen (57 cases, 81.43% of all Gram‐positives), while only two cases were bacillus. Coccus included nine micrococcus and 49 non‐micrococcus (most commonly *Staphylococcus*, 48 cases). Gram‐negative pathogens (11 cases, 15.71% of positive cultures) were most commonly *Klebsiella pneumoniae* (five cases, 7.14% of all positive cultures) and also included *Acinetobacter baumannii* (two cases), *Pseudomonas* (three cases), and others (one case).

**TABLE 4:** Types of organisms causing infection

Organisms	Number	Percent (%)
Culture‐positive	70	35.714
Gram‐negative	11	
Acinetobacter baumannii	2	1.020
Klebsiella pneumoniae	5	2.551
Pseudomonas	3	1.531
Other	1	0.510
Gram‐positive	59	
Bacillus	2	1.020
Coccus	57	
Micrococcus	8	4.082
Non‐micrococcus	49	
Enterobacter	1	0.510
Staphylococcus	48	
Epidermidis	19	9.694
Auricularis	1	0.510
Saprophyticus	1	0.510
Aureus	1	0.510
Haemolyticus	9	4.592
Capitis	4	2.041
Other	13	6.633
Culture‐negative	126	64.286
Total	196	100.000

### 3.5 Antibiotic resistance in pathogens isolated from CSF of patients in the case group

All positive isolates were fully resistant to aztreonam, cefazolin, and benzylpenicillin, and susceptible to linezolid and minocycline. A majority of isolates were resistant to cefuroxime (90.91%), penicillin (89.58%), ampicillin (83.33%), ampicillin/sulbactam (81.82%), ceftriaxone (81.82%), erythromycin (74.00%), and biapenem (71.43%). The isolates were highly susceptible to tigecycline (1.85%), vancomycin (2.04%), rifampicin (6.25%), nitrofurantoin (11.86%), amikacin (18.18%), moxifloxacin (20.41%), and gentamicin (26.67%). In addition, the remaining resistant rate varied from 30.61% to 63.64% (Table 5).

**TABLE 5:** Antibiotic resistance in pathogens isolated from CSF of patients in the case group

Antibiotics	Total number	Resistant number	Resistant rate (%)
Aztreonam	8	8	100.00
Cefazolin	11	11	100.00
Benzylpenicillin	1	1	100.00
Cefuroxime	11	10	90.91
Penicillin	48	43	89.58
Ampicillin	12	10	83.33
Ampicillin/sulbactam	11	9	81.82
Ceftriaxone	11	9	81.82
Erythromycin	50	37	74.00
Biapenem	7	5	71.43
Cefotetan	11	7	63.64
Cefepime	11	7	63.64
Piperacillin	11	7	63.64
Cefoxitin	48	30	62.50
Clindamycin	49	27	55.10
Meropenem	11	6	54.55
Imipenem	11	6	54.55
Ceftazidime	11	5	45.45
Tobramycin	11	5	45.45
Piperacillin/tazobactam	11	5	45.45
Ciprofloxacin	60	26	43.33
Levofloxacin	58	25	43.10
Cefoperazone/sulbactam	10	4	40.00
Polymyxin B	3	1	33.33
Tetracycline	49	15	30.61
Gentamicin	60	16	26.67
Moxifloxacin	49	10	20.41
Amikacin	11	2	18.18
Nitrofurantoin	59	7	11.86
Rifampicin	48	3	6.25
Vancomycin	49	1	2.04
Tigecycline	54	1	1.85
Linezolid	49	0	0.00
Minocycline	2	0	0.00

## 4 DISCUSSION

Intracranial infection, one of the most severe postoperative infections after craniotomy, is a difficulty in neurosurgical treatment (Shi et al., 2017). In the present study, 2,174 patients who underwent craniotomy were enrolled; among them, 196 patients were infected with intracranial infection during hospitalization with a rate of 9.02%. The rate was similar to the previous study, which reported that the incidence of intracranial infection after craniotomy was from 1.4% to 9.5% (Shi et al., 2017). However, the infection rate was significantly higher than other reports, which might be due to the patient's severe condition with a variety of underlying diseases. Although antibiotics and surgical drainage can be used to treat intracranial infection, the BBB provides an obstacle for drug delivery to central nervous system, and CSF only can be drained a little through lumbar puncture. Therefore, it is important to determine the risk factors for intracranial infection after craniotomy. In this study, the results indicated gender, age, CSF leakage, ASA score, surgical duration, and others were the risk factors, which have been reported in previous studies (Fang et al., 2017; Kourbeti et al., 2007, 2015; Lin, Zhao, & Sun, 2015), while season and postoperative oral infection were firstly taken into account in this study.

Previously, Adaaquah et al have reported that season is related to the variation of arterial blood pressure (Adaaquah et al., 2018). In this study, *Staphylococci* isolated from infected CSF sample were identified as the major pathogen. *Staphylococci*, as the main infectious bacteria of intracranial infection, could widely distribute on human skin, especially in spring and summer, due to the moister and hotter weather (Kourbeti et al., 2015; Zhan et al., 2014). Furthermore, patients who underwent surgery in autumn were more likely to infect with intracranial infection than those who underwent surgery in spring (OR = 2.866, 95% CI: 1.592–5.159). Therefore, in this study, season was firstly explored, and it might be an independent risk factor for intracranial infections, which might recommend that patients after craniotomy should live in a constant temperature and humidity ward. The exact mechanism of seasonal factors for intracranial infection is still further research.

In this study, postoperative oral infection was also identified as an independent risk factor for intracranial infection after craniotomy. Moazzam et al reported bacteria could adversely disseminate from oral infection to intracranial infection (Moazzam et al., 2015). Previous studies have reported that 1,200 different types of microbes were isolated from the human mouth (Corson, Postlethwaite, & Seymour, 2001; Dewhirst et al., 2010). Microorganism in the mouth could enter into the cranial vault via blood, venous drainage, inoculation, or lymphatic drainage (Moazzam et al., 2015). Furthermore, Andersen and Carpenter et al showed the central nervous system (CNS) infection correlated with oral infection, as 32%–60% of brain abscesses have been shown to be polymicrobial (Andersen & Horton, 1990; Carpenter, Stapleton, & Holliman, 2007).

Furthermore, other risk factors for intracranial infections were also explored, such as gender, age, and surgical duration, but there was no consensus on why these factors were risk factors for intracranial infections (Kourbeti et al., 2015; Lin et al., 2015). This study showed men were 1.775 times likely to have intracranial infections than women after craniotomy, which might be because men were more likely to smoke and drink alcohol than women. Farrokhi et al have reported smoking could increase the risk of infection during deep brain stimulation surgery (Farrokhi et al., 2019). More young people choose neurosurgery than the elderly, due to the limitations of income, referral system, and religious beliefs for the elderly (Cassir et al., 2015; Inoue et al., 2015). In this study, the ultimately enrolled people were relatively young (48.87 ± 16.21 years), and patients with the age ≤45 years were more susceptible to be infected with intracranial infections (OR = 2.738, 95% CI: 1.737–4.318) than those >45 years old, which was similar to the report of Zhan et al. (2014) but contrary to the report of Fang et al. (2017). Some studies have confirmed that longer operation increased the invasion of pathogens into skull and increased the risk of damaging brain tissue (Golebiowski, Drewes, Gulati, Jakola, & Solheim, 2015; Shi et al., 2017). Yao and Liu found surgical time was an independent risk factor for intracranial infections after craniotomy through analyzing 94 multiple trauma patients treated with craniotomy (Yao & Liu, 2019). All the above similarly to this study, surgical duration (≥4 hr) was identified as an independent risk factor for intracranial infections.

In the present study, there were 70 positive cultures among 196 CSF cultures, with a positive rate of 35.71%. Shi et al. reported a higher rate of positive intracranial infection results (42.7%), but patients were all with brain tumors (Shi et al., 2017). Antibiotic resistance in pathogens isolated from CSF was tested, the finding showed that 69.39% positive cultures were sensitive to tetracycline. Kourbeti et al have reported that one hundred percent of the pathogens isolated from meningitis and ventilator‐associated pneumonia (VAP) was sensitive to tetracycline (Kourbeti et al., 2015). Different from previous studies, this study found microorganisms isolated from CSF broth were most sensitive to linezolid and minocycline. In addition, the isolates (98.15%) carried a high degree of sensitive to tigecycline. The results could be served as a guide for clinical medications so that clinicians could prescribe drugs more accurately for patients with intracranial infection.

There were two limitations in the present study. The enrolled patients in both case and control groups were all from the Second Hospital of Hebei Medical University, and the samples were not randomly selected to perform craniotomy, which will produce Berkson's bias. Thus, patients in multiple hospitals were needed to reduce Berkson's bias in the future study. Besides, the sample size of our study was still smaller than the multicenter ones and the confidence interval was narrow. In our future study, we will collect larger sample size to confirm the study.

In conclusion, it was of great significance to explore risk factors for intracranial infection after craniotomy in order to timely prevent it. In this study, surgical season and postoperative oral infection were firstly determined as the new risk factors for intracranial infection. Meanwhile, microorganisms and its antibiotic resistance isolated from intracranial infections were determined. A better understanding of the targeted risk factor, microorganisms, and its antibiotic resistance of intracranial infection after craniotomy could encourage us to adopt more favorable prevention, treatment, and strategies, which contribute to the prognosis of patients.

## CONFLICT OF INTEREST

All authors have no conflicts of interests to disclose.

## AUTHOR CONTRIBUTIONS

Li‐Yi Wang: Conception and design. Xu‐Hua Cao: Administrative support. Li‐Ke Shi: Provision of study materials or patients. Zhi‐Zhao Ma: Collection and assembly of data. Yue Wang: Data analysis and interpretation. Yan Liu and Xu‐Hua Cao: Manuscript writing. All authors: Final approval of the manuscript.

## ETHICAL APPROVAL

This study has been approved by Research Ethics Committee of the Second Hospital of Hebei Medical University (2018‐R084).

## Supporting information

Table S1

Table S2

## Data Availability

The data that support the findings of this study are available on request from the corresponding author. The data are not publicly available due to privacy or ethical restrictions.
