# Supplementary material for: Risk factors for intracranial infection after craniotomy: A case–control study
Source: Brain Behav. 2020 May 18;10(7):e01658. doi: 10.1002/brb3.1658 (PMC7375057; doi:10.1002/brb3.1658)
Supplement: Supplementary file 1 — Table S1 [file BRB3-10-e01658-s001.docx]

**Supplementary Table1** Comparison of demographic characteristics between missing value and non-missing value

| Variable | Group | Age (years) | | |  | Sex | | | |
| --- | --- | --- | --- | --- | --- | --- | --- | --- | --- |
|  |  | x ± s | F | *P* |  | Male (n) | Male (%) | *χ^2^* | *P* |
| HGB | MV (n = 35) | 45.54 ± 18.16 | 1.568 | 0.211 |  | 20 | 57.14 | 0.911 | 0.340 |
|  | NMV (n = 553) | 49.08 ± 16.07 |  |  |  | 270 | 48.82 |  |  |
| RBC | MV (n = 35) | 45.54 ± 18.16 | 1.568 | 0.211 |  | 20 | 57.14 | 0.911 | 0.340 |
|  | NMV (n = 553) | 49.08 ± 16.07 |  |  |  | 270 | 48.82 |  |  |
| hsCRP | MV (n = 46) | 47.11 ± 17.35 | 0.588 | 0.443 |  | 28 | 60.87 | 2.663 | 0.103 |
|  | NMV (n = 542) | 49.02 ± 16.12 |  |  |  | 262 |  |  |  |
| ALBP | MV (n = 42) | 45.14 ± 17.51 | 2.396 | 0.122 |  | 24 | 57.14 | 1.107 | 0.293 |
|  | NMV (n = 546) | 49.16 ± 16.09 |  |  |  | 266 |  |  |  |

MV: missing value; NMV: non-missing value; HGB: hemoglobin; RBC: red blood cell count; hsCRP: high-sensitivity C-reactive protein; ALBP: albumin

Explanation:

The missing data in postoperative serum HGB, RBC, hsCRP and ALBP were 35, 35, 46 and 42, respectively. The baseline between every kinds of missing value group and non-missing value group were comparable with P value above 0.05. Meanwhile, we use expectation maximization (EM) in missing value analysis of SPSS 22.0 (IBM Corporation, New York, NY) to replace missing values. The P value of Little’s MCAR test was 0.000 which represented the missing values were missing at random.
