# Supplementary material for: Risk factors for intracranial infection after craniotomy: A case–control study
Source: Brain Behav. 2020 May 18;10(7):e01658. doi: 10.1002/brb3.1658 (PMC7375057; doi:10.1002/brb3.1658)
Supplement: Supplementary file 2 — Table S2 [file BRB3-10-e01658-s002.docx]

**Supplementary Table 2** Variable and assignment of logistic regression

| Variable | Assignment |
| --- | --- |
| Gender | Women = 0, men = 1 |
| Age (years) | > 45 = 0, ≤ 45 = 1 |
| Hypertension | No = 0, yes = 1 |
| Trauma surgery | No = 0, yes = 1 |
| Tumor surgery | No = 0, yes = 1 |
| Surgical season | Spring = 0, Summer = 1, Autumn = 2, Winter = 3 |
| Surgical duration ≥ 4h | < 4h = no, ≥4h = yes |
| Intraoperative blood loss ≥ 400ml | < 400 ml = no, ≥400 ml = yes |
| Postoperative oral infection | No = 0, yes = 1 |
| Postoperative coma | No = 0, yes = 1 |
| Postoperative RBC > normal value | No = 0, yes = 1 |
| Intracranial infection | No = 0, yes = 1 |

RBC: red blood cell count
